# Ring-Polymer Instanton Tunneling Splittings of Tropolone and Isotopomers using a Δ-Machine Learned CCSD(T) Potential: Theory and Experiment Shake Hands

**DOI:** 10.1021/jacs.3c00769

**Published:** 2023-04-20

**Authors:** Apurba Nandi, Gabriel Laude, Subodh S. Khire, Nalini D. Gurav, Chen Qu, Riccardo Conte, Qi Yu, Shuhang Li, Paul L. Houston, Shridhar R. Gadre, Jeremy O. Richardson, Francesco A. Evangelista, Joel M. Bowman

**Affiliations:** † Department of Chemistry and Cherry L. Emerson Center for Scientific Computation, Emory University, Atlanta, Georgia 30322, United States; ‡ Laboratory of Physical Chemistry, ETH Zürich, 8093 Zürich, Switzerland; ¶ RIKEN Center for Computational Science, Kobe 650-0047, Japan; § Organisch-Chemisches Institut, University of Münster, 48149 Münster, Germany; ∥ Independent Researcher, Toronto M9B0E3, Canada; ⊥ Dipartimento di Chimica, Università Degli Studi di Milano, Via Golgi 19, 20133 Milano, Italy; # Department of Chemistry, Yale University, New Haven, Connecticut 06520, United States; @ Department of Chemistry and Chemical Biology, Cornell University, Ithaca, New York 14853, United States; ∇ Department of Chemistry and Biochemistry, Georgia Institute of Technology, Atlanta, Georgia 30332, United States; △ Department of Scientific Computing, Modelling and Simulation, Savitribai Phule Pune University, Pune 411 007, India

## Abstract

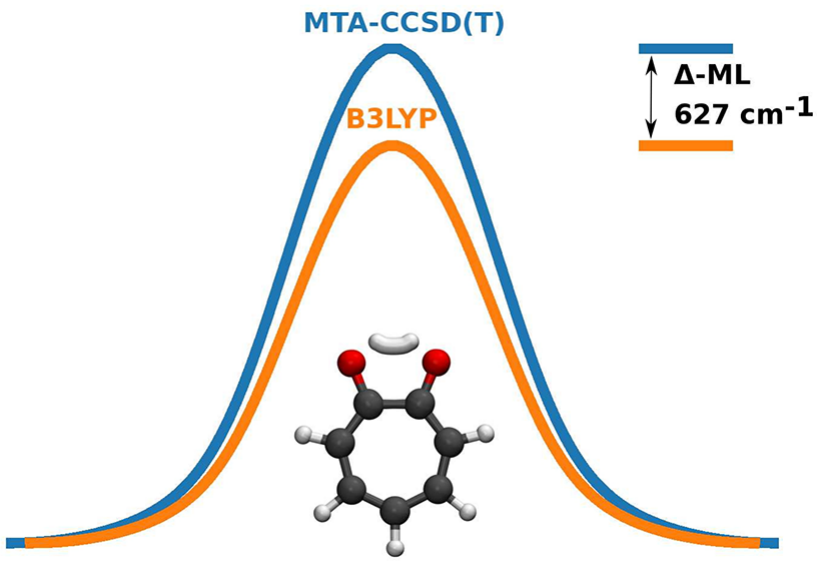

Tropolone, a 15-atom cyclic molecule, has received much interest both experimentally and theoretically due to its H-transfer tunneling dynamics. An accurate theoretical description is challenging owing to the need to develop a high-level potential energy surface (PES) and then to simulate quantum-mechanical tunneling on this PES in full dimensionality. Here, we tackle both aspects of this challenge and make detailed comparisons with experiments for numerous isotopomers. The PES, of near CCSD(T)-quality, is obtained using a Δ-machine learning approach starting from a pre-existing low-level DFT PES and corrected by a small number of approximate CCSD(T) energies obtained using the fragmentation-based molecular tailoring approach. The resulting PES is benchmarked against DF-FNO-CCSD(T) and CCSD(T)-F12 calculations. Ring-polymer instanton calculations of the splittings, obtained with the Δ-corrected PES are in good agreement with previously reported experiments and a significant improvement over those obtained using the low-level DFT PES. The instanton path includes heavy-atom tunneling effects and cuts the corner, thereby avoiding passing through the conventional saddle-point transition state. This is in contradistinction with typical approaches based on the minimum-energy reaction path. Finally, the subtle changes in the splittings for some of the heavy-atom isotopomers seen experimentally are reproduced and explained.

## Introduction

Proton (H atom) transfer reactions, which often involve quantum-mechanical tunneling, are pervasive phenomena in many chemical and biological processes.^1^ There is also evidence that heavy-atom tunneling can play an important role in organic reactions.^2−7^ Tunneling splits the lines in the spectrum of a molecule, leaving a clear signature that can be probed with a variety of experimental techniques. Despite this, there is a rather limited set of molecules to which theoretical quantum-dynamical studies have been applied. The molecule which has received most theoretical attention is malonaldehyde, with numerous approximate approaches applied over several decades.^8−17^ The first spectroscopically accurate, CCSD(T)-based potential energy surface (PES) for malonaldehyde was reported in 2008.^18^ Diffusion Monte Carlo (DMC) calculations using this PES obtained agreement with experiment for the ground-state splitting for H and D transfer (21.6 cm^–1^ exptl and 22–23 cm^–1^ theory for H and 2.9 cm^–1^ exptl and 2–4 cm^–1^ theory for D),^18^ and subsequent quantum MCTDH calculations using that PES validated those results.^19,20^ A more recent CCSD(T)-based PES has also been reported, and good agreement with experiment was obtained using guided DMC calculations.^21^ With these accurate calculations on accurate PESs, tests of approximate methods followed. One, the very simple Q_im_-path method,^12^ did provide reasonable accuracy by predicting 26 cm^–1^ for H atom and 4.6 cm^–1^ for D atom transfer. The more sophisticated semiclassical ring-polymer instanton (RPI) method^13,22^ provided even more accurate results, i.e., 19.3 and 2.7 cm^–1^ for H and D atom transfer, respectively.^14^ The accuracy of RPI was also illustrated for a 10-atom formic acid dimer using an accurate CCSD(T)-based PES,^23^ where the calculated splitting^24^ of 0.014 cm^–1^ is in remarkably good agreement with the most up-to-date experimental value of 0.011 cm^–1^.^25^

Tunneling splittings for larger molecules using full-dimensional PESs are just beginning to appear. Some of us reported a DMC calculation of the ground-state tunneling splitting of 15-atom acetylacetone, first using an MP2-based permutationally invariant polynomial (PIP) PES.^26,27^ The splitting for H atom transfer is 160 cm^–1^. We noted in that work that the barrier for H atom transfer on the MP2-based PES is significantly lower than the accurate CCSD(T) barrier. In response we applied our recent Δ-maching learning (ML) approach^28^ to develop a Δ-ML CCSD(T) PES,^29^ on which the H atom splitting is 32 cm^–1^. Unfortunately, there are no experimental measurements of this splitting, as far as we know. We note that Δ-ML for potentials is an active research area.^17,30−34^ Of particular relevance to this paper is the recent one^17^ using transfer learning to bring an MP2-based PES for malonaldehyde to the CCSD(T) level, which focused in particular on the region of configuration space probed by the instanton. In that work, a concrete procedure for selecting new points at which high-level calculations should be carried out for training/learning was proposed.

**Figure 1:**
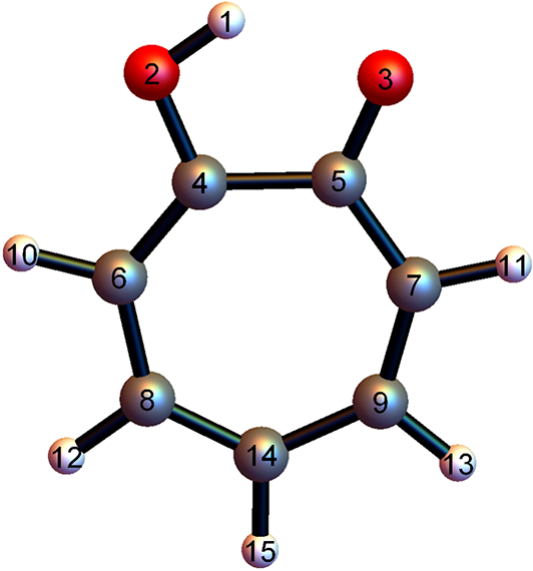
Numbering scheme used for tropolone. H, C, and O atoms are white, gray, and red, respectively. This scheme is taken from ref (52), where details, which are irrelevant for the present work, are given.

There is substantial experimental data for tropolone,^35−46^ but as far as we are aware there is no previous full-dimensional theoretical study. The 15-atom molecule is depicted in Figure 1, along with the atom numbering scheme we use below. As in the smaller and intensively studied malonaldehyde molecule, one hydrogen in tropolone tunnels through the barrier of a double well, resulting in a splitting of vibrational levels.^1^ The splittings are smaller than those for malonaldehyde. i.e., 0.97 cm^–1^ (tropolone) vs 21 cm^–1^ (malonaldehyde) for H atom tunneling. These detailed measurements are strong motivation for a first-principles study, i.e., an accurate potential energy surface as well as a rigorous full-dimensional approach for determining the splitting. It should be noted that full dimensional quantum approaches to obtain the small splittings in tropolone are either not precise enough (in the case of DMC) or not feasible for this large molecule. In this paper, we therefore apply the RPI method to compute the tunneling splitting of tropolone and a number of its isotopomers. We note that there have been several previous theoretical approaches to this problem;^47−50^ these were all highly approximate, starting with model potentials. A recent semiclassical calculation of splitting in tropolone is a notable step forward.^51^ In that work a one-dimensional version of semiclassical VPT2 theory was used to obtain splittings of H and D transfer for tropolone. The approach required using a quartic force field expanding around the saddle point configuration and then replacing the MP2 barrier height by a CCSD(T) one at the MP2 saddle point. Agreement with the experiment for H transfer was excellent but off by a factor of 2 for the D transfer. Prior to our work,^52^ this paper, published in 2020, represented the state of the art for the tropolone tunneling splittings. As such, it is worth quoting the following from that paper: “For this system, with 15 atoms, a full-dimensional treatment using a large basis set is prohibitively expensive.” Here we achieve this objective.

It is clear that a machine-learned PES for tropolone needs to be trained on a large data set. So using a low-level method that provides gradients as well as energies is an approach (perhaps the only approach) that is feasible. Indeed, the previous DFT-based PES was a fit using 6601 energies and the corresponding 297 045 gradient components.^52^ The choice of geometries is described in detail in ref (53). Briefly, 3300 geometries were taken using every 10th point of *ab initio* molecular dynamics trajectories started from the global minimum at energies of 4000, 10 000, 20 000, 30 000, and 40 000 cm^–1^ or from Conformer I (where the OH has pointed away from the remaining oxygen) at an energy of 6000 cm^–1^. The additional 3301 geometries were taken from calculations on grids centered on the global minimum or the H-transfer saddle point. The permutational symmetry of the tropolone PES (including the double well) was treated using PIPs. However, as expected, this DFT PES is not accurate enough for our purposes, as the splittings are known to be very sensitive to the height and width of the barrier along the tunneling path. It can, however, be used as the basis of a Δ-ML approach^28^ to reach near CCSD(T) quality.

Ideally, we would learn the difference between the DFT and the “gold standard” CCSD(T) level. However, this is not feasible (for us) owing to ∼*N*^7^ scaling, where *N* is the number of basis functions of CCSD(T) theory. For tropolone, a single-point calculation with an aug-cc-pVTZ basis using Molpro^53^ is prohibitively expensive on our cluster. (This conclusion is based on timings for approximate CCSD(T) calculations given below.) Therefore, we use the fragmentation-based molecular tailoring approach (MTA), developed by Gadre and co-workers,^54^ to obtain approximate CCSD(T) energies. The MTA has been shown to give energies with a typical accuracy of ∼1 millihartree (1 mH) compared with the respective full calculation energy with an advantage in the wall clock time by a factor typically between 3 and 5.^54^ Several test cases for benchmarking the accuracy and efficiency of MTA vis-à-vis the respective full calculations have recently been reported by Khire et al.^55^ These test cases included the minimum and transition-state geometries of acetylacetone, *cis*- as well as *trans*-*N*-methylacetamide, and tropolone. Additionally, two more geometries for each of the above systems were also employed for benchmarking purposes. Apart from that, the performance of MTA was critically assessed for generating the CCSD(T)/aVTZ level PES for the 15-atom acetylacetone molecule using 550 basis functions.^55^ For the H-transfer barrier height the MTA-CCSD(T) value was within 0.17 kcal/mol of the CCSD(T)/aVTZ one. For benchmarking purposes, we also perform a small number of CCSD(T)/aVTZ and CCSD(T)-F12/aVTZ calculations for tropolone.

The outline of this paper is the following. In the next section, we briefly review the Δ-ML method for the PES, followed by the molecular tailoring approach. The section concludes with a review and details of the RPI method and calculations. Following that, the Results and Discussion section starts with the precision and details of the new Δ-ML PES. The RPI tunneling splittings are presented for H atom transfer and nine isotopomers for both the original DFT-based PES and the new Δ-ML PES. Estimates of the fitting error are made using direct MTA-CCSD(T) energies along the instanton path. These energies are not included in the training data for the fit. This provides a reasonable test of the sensitivity of the splittings obtained with the new PES. Comparisons with experiments are made and discussed. A summary and conclusions are given in the final section.

## Methods and Calculation Details

### Δ-Machine Learned Potential

Δ-Machine learning^31,56^ is a general method to bring a property such a PES trained on an efficient ab initio method, such DFT, to the gold standard CCSD(T) level. Here we use the Δ-ML method proposed and tested extensively by some of us.^28,29,34^ The expression for the Δ-ML PES is given by
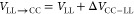
1where *V*_LL→CC_ is the corrected PES, *V*_LL_ is a PES fit to low-level B3LYP/6-31+G(d) energies and gradients (from previous work),^52^ and Δ*V*_CC–LL_ is the correction PES which is a fit to the difference in high-level and low-level energies only (i.e., without gradients).^28^*V*_LL_ and Δ*V*_CC–LL_ are represented in a basis of PIPs^57−59^ with linear coefficients that are determined using standard linear algebra methods.

For the correction PES, Δ*V*_CC–LL_, a data set of 2044 MTA-CCSD(T) energies was used for the fit. These were obtained at a subset of the 6604 geometries used for DFT PES.^52^ The fitting basis for Δ*V*_CC–LL_ uses the same symmetry as the one for the DFT PES and has a maximum order of 2. This results in a basis with only 252 polynomials. This small number is largely a consequence of the small data size of MTA-CCSD(T) energies. Nevertheless, the MTA-CCSD(T) energies span a range that extends more than 20 000 cm^–1^ above the global minimum. In the Results and Discussion section we examine the fidelity of the fit to Δ*V*_CC–LL_ and test the accuracy of the corrected PES by comparing to direct MTA-CCSD(T) energies along the instanton path.

### Molecular Tailoring Approach

Within MTA, the large molecule under consideration is divided notionally into two or more overlapping fragments. The electronic structure calculations are then run only on the fragments rather than the whole molecule. The desired electronic property, *P* (being the energy in the present work), of the molecule is then estimated by adding and subtracting the respective properties of the *n* fragments using the set inclusion–exclusion principle (cf. eq 2) in an appropriate sense.
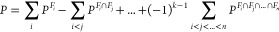
2Here, *P*^*F*_*i*_^ denotes the electronic property of the *i*^th^ main fragment, while *P*^*F*_*i*_∩*F*_*j*_^ represents the property *P* of the overlap species between the fragments *F*_*i*_ and *F*_*j*_. The term *k* is the order of overlap between the fragments. For more details, see ref (54).

MTA results have an error compared to a full calculation (FC), due to the neglect of interactions between distant atoms. In order to correct this error, a grafting procedure^54^ was proposed, wherein the correction is estimated by doing a couple of overhead computations: (i) MTA calculations at an appropriate lower basis set (LB) maintaining the fragmentation scheme and level of theory unaltered; (ii) FC calculation at same of level of theory executed in conjunction with the LB. The difference between these two computations is added (grafted) to the property estimated by MTA at a higher basis (HB) set; see eq 3.
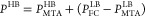
3Here, *P*^HB^ represents the electronic property of the whole molecule after adding the grafting correction. *P*_MTA_^HB^ is the property computed by the MTA procedure employing the HB in conjunction with eq 2. The terms *P*_FC_^LB^ and *P*_MTA_^LB^ are the property values by doing the FC and MTA computations, respectively, at the LB.

**Figure 2:**
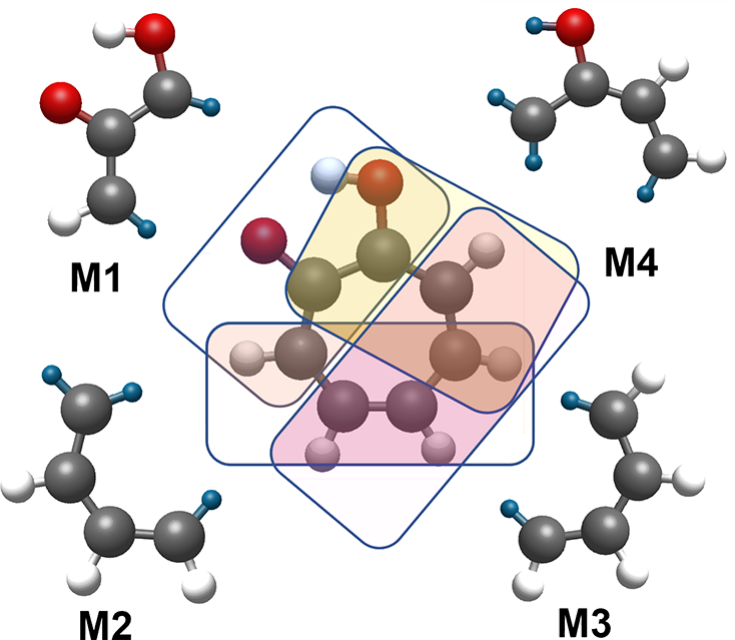
Fragmentation scheme implemented for MTA computations on the tropolone molecule. Four main fragments are depicted as M1, M2, M3, and M4. The red, gray, and black balls are oxygen, hydrogen, and carbon, respectively. The blue balls are dummy hydrogen atoms. See text for details.

For this purpose four fragments M1, M2, M3, and M4 comprising 9, 10, 10, and 11 atoms, respectively, are constructed. Four overlapping fragments are generated from these fragments. As per the MTA guidelines, valences of each atom are satisfied by adding a dummy hydrogen atom. The fragmentation scheme, displayed in Figure 2, is applied to all the geometries of tropolone.

The cc-pVTZ basis set is used as the LB for grafting correction; see eq 3. The ab initio package^60^ Gaussian 16 with “frozen core” as the default option is used at the back end while executing the MTA. An Intel Xeon processor-based single computational node with 16 cores is used for the energy estimation. The typical wall-clock timings for performing the FC and MTA runs are 15.8 and 4.3 h, respectively. The approximate disk requirement for running an FC job at the CCSD(T)/aVTZ level is ∼170 GB. However, MTA accomplishes this computation using only ∼38 GB of disk space. Thus, MTA brings tropolone into the realm of computational tractability at the CCSD(T) level with accuracy and efficiency with modest hardware.

Subsequent to performing MTA-CCSD(T) calculations and using them to obtain the new corrected PES, the energy along the instanton path was computed with the density-fitted frozen natural orbital CCSD(T) [DF-FNO-CCSD(T)] method. A brief description of the method and limited calculations are given in the Supporting Information.

### Ring Polymer Instanton Theory

Instanton theory has become a well-established method for calculating the tunneling splitting of a degenerate rearrangement.^13,22,61^ Recently, it has also been extended to study asymmetric double-well systems in which isotopic substitution makes the two wells slightly nondegenerate.^14^ To evaluate the level splitting, we require two quantities. First,  is the contribution due to the asymmetry of the double well, which can easily be evaluated by simply measuring the zero-point energies of the left and right wells,  and *E*_r_. In this work, these energies are calculated within the harmonic approximation, although in principle anharmonic contributions can also be included in cases where the harmonic approximation is not sufficient.^62^ Second, ℏΩ is the contribution due to quantum tunneling effects. The experimental observable is the level splitting, which can be predicted from the formula . In the case of a symmetric double well (where *d* = 0), the splitting is simply given by Δ = 2ℏΩ.

In this work, we evaluate Ω using semiclassical instanton theory, a method that describes quantum tunneling through the barrier, but at a highly reduced computational cost compared to exact quantum-mechanical methods.^13,14,61,63−66^ This approach relies on the properties of a minimum-action pathway, known as the “instanton”, which connects the two wells. The tunneling contribution is then evaluated as
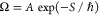
4where *S* is the action along the instanton path and *A* corresponds to the fluctuations around this path, as defined in previous work.^61^ RPI theory approximates the fluctuations to second order and thus neglects anharmonicity perpendicular to the tunneling path. Anharmonicity along the path is accurately captured, as long as the barrier height is significantly larger than the splitting,^13^ which is certainly the case in tropolone. Based on previous experience with the chemically similar malonaldehyde molecule,^14^ we can expect the instanton approximation to predict splittings within about 10% of the quantum-mechanical result for the same PES.

In RPI theory the instanton is discretized into *N* ring-polymer beads, wherein each bead is a single snapshot of the molecular geometry along the tunneling path. In practice, we have to converge the calculation in the limits *N* → ∞ and *T*_eff_ → 0, where *T*_eff_ is an effective temperature. In this work, we carried out instanton optimizations as described in ref (61), achieving convergence with  beads at . The instanton optimization was carried out separately for each isotopomer because, although the pathways are similar, they are not identical. This procedure was carried out for both the Δ-ML CCSD(T)-quality PES introduced in this work and the previous PIP-PES trained purely with DFT data.^52^

## Results and Discussion

### Δ-ML CCSD(T) PES (*V*_LL→CC_)

The correction PES, Δ*V*_CC–LL_, is fit to the difference in electronic energies. As expected based on previous work,^28,34^ the difference is small and slowly varying compared to the electronic energy. Indeed, this is a necessary condition for this Δ-ML method to work efficiently. In the present case, the energy differences are several thousand wavenumbers. A scatter plot of the energy differences is given in the Supporting Information. Note that we reference Δ*V*_CC–LL_ to the minimum of the difference between the CCSD(T) and DFT energies (roughly 6000 cm^–1^). As seen there, the data set includes high-energy configurations for which Δ*V*_CC–LL_ is large. These high-energy configurations are irrelevant in this study, as the instanton only probes the barrier region; however, they do permit the final PES to be extended to high energies without many “holes” and so can in principle be used in quantum calculations such as diffusion Monte Carlo, VSCF/VCI, MCTDH, etc. Also it is seen that Δ*V*_CC–LL_ is not as strongly varying as *V*_LL_ with respect to the nuclear configuration, as expected. The fit uses a maximum polynomial order of 2 with the same reduced permutational symmetry as used for the DFT-based low-level PES.^52^ This results in a basis of 252 PIPs and thus 252 linear coefficients. The PIP basis to fit this PES is generated using our monomial symmetrization software, denoted MSA.^67−69^ The correction PES is added to the low-level PES to give the final corrected PES, *V*_LL→CC_. A plot of this PES vs corresponding direct MTA-CCSD(T) energies for the training set of 2044 points is shown in the Supporting Information, where overall excellent precision is seen. The RMS differences between the *V*_LL→CC_ and direct MTA-CCSD(T) energies for the training data set are 80 cm^–1^ for energies up to 10 000 cm^–1^, 95 cm^–1^ for energies up to 20 000, and 105 cm^–1^ for the entire data set. Another stringent test of the precision of the corrected PES, *V*_LL→CC_, is a comparison between the predicted energies of *V*_LL→CC_ against direct MTA-CCSD(T) ones not in the training data. This is done on the instanton path on *V*_LL→CC_, and the results are shown below, following the presentation of the instanton results.

Geometry optimization and normal-mode frequency calculation of both global minimum and its H-transfer saddle point geometries were done with the Δ-ML PES, and the results are given in the Supporting Information. At the saddle point we obtain the H-transfer barrier height as 2512 cm^–1^ (7.18 kcal/mol) from this V_LL→CC_ PES, whereas the DFT PES barrier is 2061 cm^–1^ (5.89 kcal/mol).^52^ Below we make comparisons of energies at the maximum of the instanton path, which are more relevant to the RPI tunneling splittings.

Finally, note that because the PIP basis for Δ*V*_CC–LL_ is 100 times smaller than the one for *V*_LL_, the additional time to calculate Δ*V*_CC–LL_ for the corrected PES *V*_LL→CC_ is virtually zero.

### Instanton Splittings

**Figure 3:**
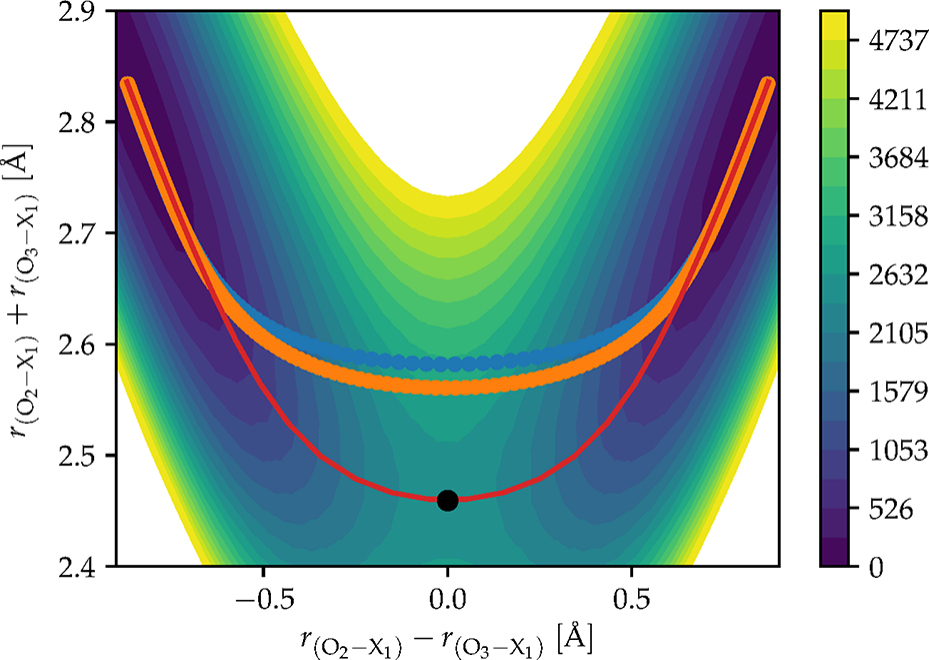
A 2D representation of the tropolone Δ-ML PES, where the energy contours are determined by a constrained optimization at each point with fixed O_2_–X_1_ and O_3_–X_1_ bond lengths (where X = H, D). The color bar indicates units of energy in cm^–1^. Here, we show two ring-polymer instantons. The parent is shown by the blue circles connected by a blue line, and the deuterated isotopomer D_1_ is shown by the orange circles connected by an orange line. The minimum-energy pathway is shown by a red line, and finally, the conventional saddle point/transition state is indicated by the black dot.

A depiction of the ring-polymer instanton pathway for tropolone is shown in the graphical abstract. Here, it can be observed that the H_1_ atom is the most heavily involved in the proton transfer process. Figure 3 shows a projection of the tunneling pathway, represented by the blue circles connected by a blue line, on a two-dimensional representation of the PES. The instanton can also be compared to the minimum-energy pathway (MEP), shown in red. One can clearly observe that the tunneling pathway “cuts the corner”,^70,71^ exploring a region of the PES far away from (and higher in energy than) the conventional saddle point transition state (represented by the black dot). Corner cutting in tropolone is similar to what was observed in malonaldehyde.^13,16^ Note this effect is not captured by the Q_im_-path approach, and indeed most approximate one-dimensional approaches, which assume that the tunneling path passes through the saddle point. The effect of corner cutting can be quantified using the action *S*/ℏ, which is 9.50 along the instanton and 15.67 along the MEP. This implies that neglecting corner cutting would underestimate the splitting by a factor of approximately 500.

Figure 3 additionally shows the instanton path for the deuterated isotopomer D_1_ in orange. It can be seen that the deuterated species cuts the corner less, such that the maximum potential along the tunneling pathway is lower and closer to the saddle point. Of course, this increases the overall path-length, which in turn increases the action to *S*/ℏ = 12.35 and therefore decreases the value of Ω.

In previous work,^14^ we quantified the contribution of each atom to the instanton tunneling pathway by evaluating the squared mass-weighted path-length, which itself is proportional to the action. What we find is that the H_1_ atom provides a dominant contribution of approximately 74% (or 77% for D_1_). The remaining H atoms contribute less than 1% so that the heavy (C and O) atoms contribute the remaining 26% or 23% (depending on whether the H_1_ atom has been deuterated).

Table 1 presents the results evaluated with instanton theory for both the Δ-ML PES developed in this work and the previous DFT PES.^52^ The experimentally obtained splittings are also shown. The splittings are also depicted in Figure 5. As seen, the results using the new Δ-ML PES are much closer to experiment than those from the DFT-based PES.

**Table 1:** Level Splittings  and the Separate Contributions from Asymmetry, *d*, and Tunneling, ℏΩ, All in cm^–1^^a^

	DFT(B3LYP) PES			Δ-ML PES				
isotopomer	*d*	ℏΩ	Δ	*d*	ℏΩ	Δ	Δ_Δ-ML_^corr^	Δ_expt_
parent	0	1.33	2.67	0	0.34	0.68	0.92	0.97^35,36^
^13^C_14_	0	1.33	2.66	0	0.34	0.67	0.91	0.97^36^
^18^O_2_^18^O_3_	0	1.27	2.54	0	0.32	0.63	0.86	0.87,^41^ 0.83^43^
D_1_	0	0.082	0.16	0	0.015	0.031	0.042	0.051^36,43^
^18^O_2_^18^O_3_ D_1_	0	0.078	0.15	0	0.014	0.028	0.038	0.043 (estimated)^43^
^18^O_2_	1.02	1.28	3.27	0.89	0.32	1.89	1.98	1.70 (estimated)^41,44^
^13^C_4_	–0.55	1.32	2.86	–0.62	0.33	1.41	1.54	0.89 (outlier)^36^
^13^C_6_	0.36	1.33	2.75	0.34	0.34	0.95	1.14	1.13^36^
^13^C_8_	0.10	1.33	2.67	0.11	0.34	0.71	0.94	0.98^36^
^13^C_8_ D_1_	0.10	0.082	0.26	0.042	0.015	0.091	0.095	

a The subscript numbers for the isotopomers indicate the atom(s) that were isotopically substituted, using the numbering from Figure 1, whereas the superscript numbers indicate the substituted atomic mass. Δ_Δ-ML_^corr^ corresponds to the splittings corrected with MTA-CCSD(T) points along the instanton path.

**Figure 4:**
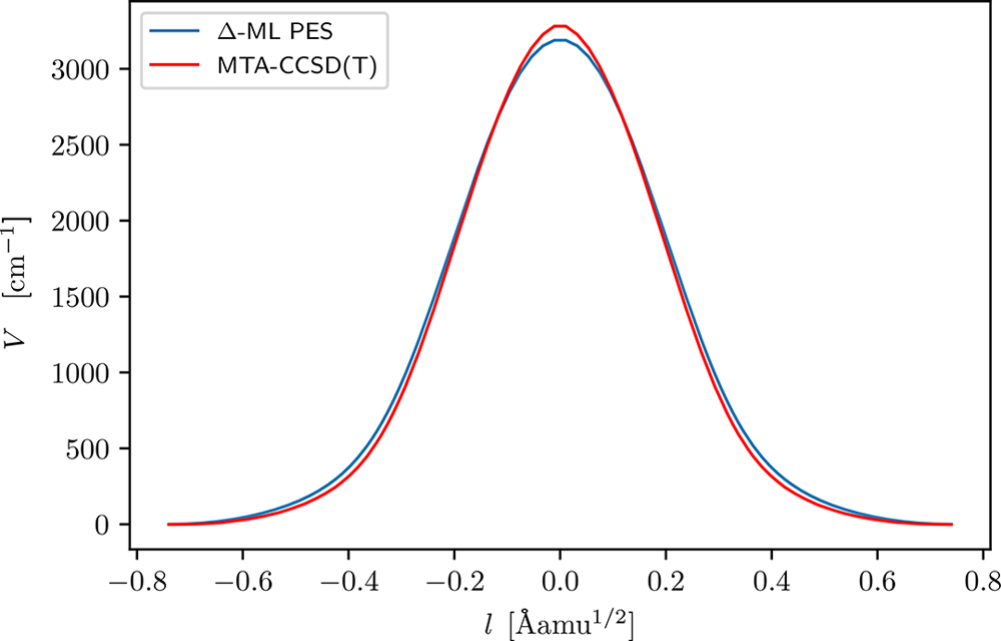
Comparison of the potential along the instanton optimized on the Δ-ML PES, and direct MTA-CCSD(T) calculations along this path, as a function of cumulative mass-weighted path-length *l* (defined in previous work, see ref (61)).

**Figure 5:**
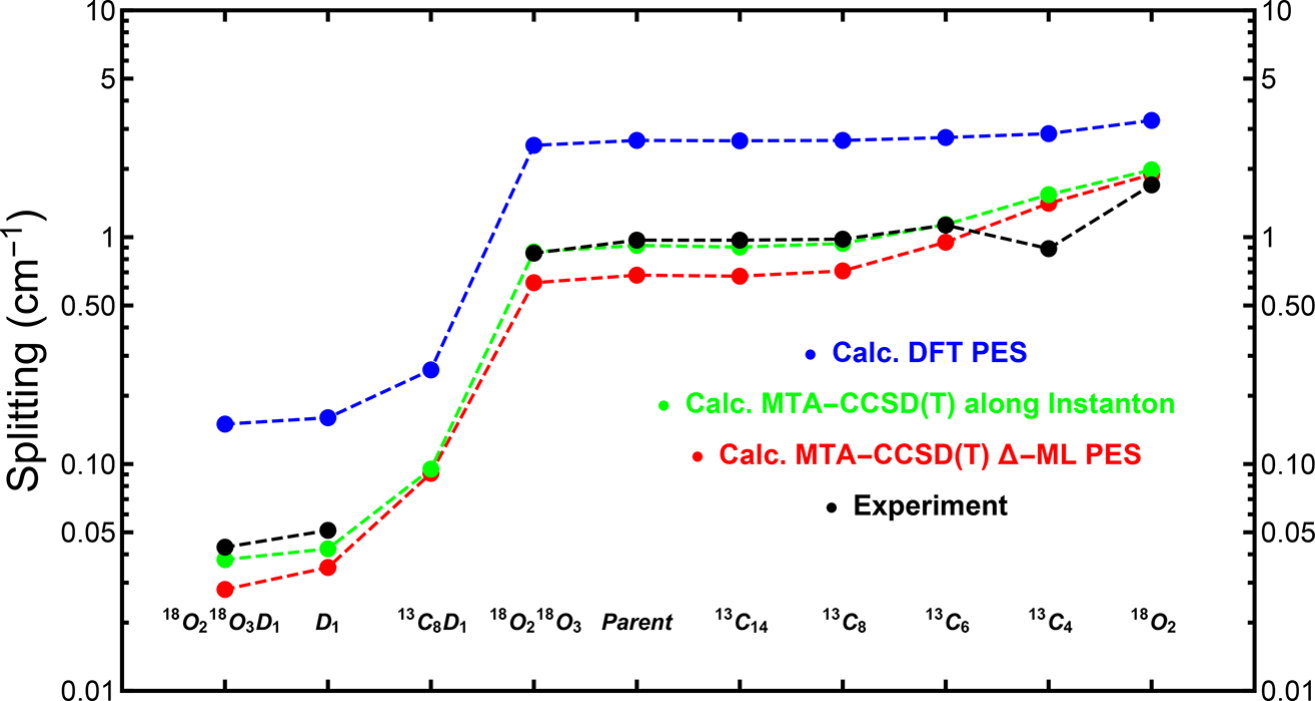
Comparison of experimental and calculated splittings. Blue, red, and green data indicate the instanton results using the DFT PES, the Δ-ML PES, and MTA-CCSD(T) calculations along the instanton path, respectively. The black data indicate the experimental results. Isotopomers are listed at the bottom, where the subscript numbers indicate the atom(s) that were isotopically substituted, using the numbering from Figure 1.

In particular, the splittings from the DFT-PES significantly overestimate the experimental measurements. The discrepancy is much more than the 10% error expected from the instanton approximation, implying that the main source of error lies with the DFT-based PES. Because of corner cutting, the usual focus on the height of the saddle point is not of direct relevance here. It is more useful to compare the energy profile along the instanton paths optimized on the DFT-based PES and the new Δ-ML PESs as shown in the graphical abstract and in the Supporting Information. The maximum energies are 2561 and 3188 cm^–1^, respectively, a difference of 627 cm^–1^ (1.80 kcal/mol). In addition to this, the DFT-PES profile is too narrow. Both these factors cause an overestimation of the splittings.

The splittings from the Δ-ML PES are in reasonable agreement with experiment, but not quite within the expected 10% error estimate. To examine the sensitivity of the splittings on the Δ-ML PES, we calculated direct MTA-CCSD(T) energies along the parent-molecule instanton path, which had been optimized on the Δ-ML PES. These results are shown in Figure 4, which are seen to be in close correspondence. However, the maximum of direct MTA-CCSD(T) energies, 3282 cm^–1^, is slightly larger than the Δ-ML PES energy, 3188 cm^–1^. This suggests that the Δ-ML PES fit could be made more precise for this path by including these points along the instanton path for the fit (similarly to ref (17)); however, see the discussion below about benchmark energies at the instanton maximum.

The direct MTA-CCSD(T) energies were used to estimate a correction to the splittings, by recalculating *S*, but assuming the same pre-exponential factor, *A* (eq 4). This approach, sometimes known as the dual-level method, has often been used in previous work.^6,16,72,73^ Details are summarized in the Supporting Information. Based on the fact that the direct MTA-CCSD(T) barrier height is slightly larger than for the Δ-ML PES, one might expect the action to increase. However, we find that the corrected action *S*_corr_ actually decreases, evidently due to the slightly smaller barrier width. This provides a correction factor of exp(−*S*_corr_ + *S*_Δ-ML_) = 1.35, which is a measure of the accuracy of the fit. We then multiply each Ω value by this factor (implicitly assuming that the correction is roughly the same for all isotopomers) to obtain the corrected splittings shown in Table 1 and Figure 5 (Δ_Δ-ML_^corr^ and ‘Calc. MTA-CCSD(T) along instanton’ respectively). The calculations using the MTA-CCSD(T) energies along the instanton path (green) give excellent splittings compared to the experiment, in most cases within the expected 10% error bars. It is probably fortuitous that the agreement is even better than this.

In the Supporting Information, we also consider a correction based on DF-FNO-CCSD(T) energies calculated along the instanton path. These give similar trends for the isotopomers but overpredict the experiments by about 0.4 cm^–1^ on average. At first sight this is surprising because the approximations behind DF-FNO-CCSD(T) are expected to be more accurate than those of MTA-CCSD(T). This is confirmed by the fact that the DF-FNO-CCSD(T) barrier is within 2 cm^–1^ of the CCSD(T)/aVTZ result, whereas MTA-CCSD(T) is almost 300 cm^–1^ higher. There is, however, evidence that the true barrier is indeed higher than that predicted by CCSD(T)/aVTZ due to the incompleteness of the basis set. This has some support in the literature for the saddle-point barrier height for malonaldehyde, the smaller sibling of tropolone. Namely, at the CCSD(T)/aVTZ level of theory the barrier height is 1362 cm^–1^ (3.89 kcal/mol),^17^ but 1430 cm^–1^ (4.09 kcal/mol) at the near complete-basis-set limit^18^ and also in more recent work using explicitly correlated methods.^21^ Subsequent to the review of the original manuscript, we verified this using (the more computationally intensive) CCSD(T)-F12/aVTZ calculations^74^ and obtain 3079 cm^–1^ at the maximum of the instanton path, which is 92 cm^–1^ higher than the CCSD(T)/aVTZ maximum. This goes some way to explaining why the instanton results corrected with direct MTA-CCSD(T) calculations are closer to experiment than those corrected with DF-FNO-CCSD(T).

We now discuss trends in the symmetric isotopomers, which are presented in the top half of Table 1. Hereafter, the discussion of results is made with reference to the Δ-ML PES, unless stated otherwise. As mentioned previously, the H_1_ atom contributes the most to the action, and thus isotopomers with a D_1_ substitution show a dramatic decrease in Δ compared to the parent isotopomer. On the other hand, the introduction of two ^18^O isotopes causes only a small, but noticeable reduction in Δ. This is mainly caused by a slight increase in the action upon substitution of ^16^O with ^18^O, indicating that these atoms are involved to some extent in the tunneling pathway. In fact the O atoms contribute 8% each to the squared mass-weighted path length along the instanton. On the other hand, the substitution of C_14_ (atom number 14 of Figure 1) with ^13^C results in a small decrease in Δ; this is because the action only slightly increases as the contribution of the C_14_ atom to the squared mass-weighted path length along the instanton is less than 1%. These same trends are reflected in the experimental results.

Next, we discuss the splittings for various asymmetric isotopomers of tropolone, which are presented in the bottom half of Table 1. The trends in the instanton results as shown in Figure 5 for the most part follow that of experiment, with perhaps just one exception. We shall now explain these trends in more detail.

As in the symmetric case, deuterating the H_1_ atom results in a massive decrease in the tunneling contribution, as H_1_ contributes the most to the action. Substitution of individual O atoms with ^18^O decreases the tunneling contribution slightly, and as for substituting the C atoms along the ring with ^13^C, the decrease in the tunneling contribution is less apparent, as these atoms contribute even less to the action (between 0.2% and 3%). In these asymmetric isotopomers, it is important to measure not only the tunneling contribution, Ω, but also the asymmetry introduced by isotopic substitution, *d*, which in turn allows one to determine the overall splitting, .

We employ a measure of localization called the mixing angle ϕ = tan^–1^(ℏΩ/*d*).^14^ This arises from the description of an effective two-level Hamiltonian for the system, for which we can write the ground- and excited-state eigenvectors as  and . The mixing angle ϕ is a measure of localization, which results from the balance between the Ω and *d* factors. When tunneling effects dominate (Ω ≫ |*d*|), the mixing angle approaches 90° and the eigenstates are maximally delocalized. However, when the asymmetry of the double well dominates (Ω ≪ |*d*|), the mixing angle approaches 0° or 180° and the ground- and excited-state wave functions are each localized to one well. For ^18^O_2_, we find a mixing angle of 20°, which corresponds to a population ratio of approximately 10:1. This is an indication that the system is quite strongly localized for the ^18^O_2_ isotopomer (as observed by Keske et al.^36^), and the splitting is dominated by the asymmetry with only minor contributions from tunneling effects. A very different story would have been found from the DFT PES, where ϕ = 51°, indicating far more mixing and a population ratio of roughly 5:3. The zero-point energies predicted by the DFT PES are not too far off from that predicted by the Δ-ML PES, with a difference of approximately 0.1 cm^–1^ for *d*. Results using the DFT-based PES, however, overestimate the role of tunneling effects and thus the mixing associated with this isotopomer.

The overall trend is that the further the isotopic substitution is from the H_1_ atom, the smaller *d* is. An interesting case is provided by the ^13^C_6_ isotopomer, wherein asymmetry and tunneling provide equal contributions to the splitting. This results in a mixing angle ϕ = 45° with the Δ-ML PES, implying a partially mixed state. The ^13^C_8_ isotopomer is the most delocalized asymmetric isotopomer studied here, with a mixing angle of ϕ = 72°. The splitting is thus mostly influenced by tunneling effects, with asymmetry providing a small, but noticeable contribution. The ^13^C_4_ isotopomer is the most localized among the ^13^C-substituted isotopomers, with ϕ = 28°.

In almost all cases, the predicted trends are in close agreement with the experimental results. However, ^13^C_4_ is a significant outlier. Unlike all other asymmetrically substituted isotopomers, the microwave experiment of Keske et al.^36^ predicts a decrease in Δ compared to the parent isotopomer. This could only be possible if Ω was significantly smaller for this particular isotopomer. This is hard to rationalize given our first-principles theoretical calculations, where we predict that Ω is almost unchanged by isotopic substitution of the O and C atoms. Note that we predict a negative value of *d* in this case, which means that Figure 1 represents the higher (rather than the lower) energy well. However, this does not affect Δ, as only the magnitude of *d* is relevant. It may be worth reassessing the experimental assignments in this case.

We also make a prediction for the isotopomer ^13^C_8_D_1_, which has not been studied experimentally. The splitting is expected to be small, as for all other observations made for isotopomers wherein the H_1_ atom has been deuterated. The substitution of ^13^C_8_ introduces a slight asymmetry, which then results in a mixing angle of ϕ = 18°. We hope that this can be confirmed in future experiments.

To summarize this section, we have studied how the splitting is affected upon isotopic substitution of various atoms. We find that substitution of the transferring proton has the largest propensity of decreasing tunneling effects, something that is also reflected in experiment. Substitution of the heavier O and C atoms affects the splitting to varying degrees. We find that, in general, isotopic substitutions of heavy atoms only very slightly reduce the effects of tunneling, i.e., the value of Ω. The magnitude of this reduction, however, depends on the contribution of the substituted atom to the action of the instanton path. For instance, substitution of O atoms with ^18^O reduces the tunneling contribution more than substitution of the C atoms with ^13^C, as the O atoms contribute more to the action. A more important factor is that the splitting additionally depends on the asymmetry introduced upon isotopic substitution, which is quantified by *d*. Thus although isotopic substitutions may not significantly affect the tunneling contribution, Ω, they can have a dramatic effect on *d* and hence the splitting, Δ, and the mixing angle, ϕ. We find that the mixing angle can vary strongly based on (i) the atom substituted and (ii) the position of the isotopic substitution (i.e., how close it is to the tunneling proton). Our findings are for the most part similar to those of Keske et al.,^36^ wherein they surmise that the states become increasingly localized depending on the isotopic substitution. However, our interpretation is also subtly different in the sense that we do not conclude that tunneling is quenched, but instead, it is mixing (and, in turn, the relative contribution of tunneling toward the overall splitting) that is quenched.

## Summary and Conclusions

We reported a Δ-machine learned potential energy surface for tropolone which is of near CCSD(T) quality. This agreement was achieved using the efficient molecular tailoring method, used to correct an earlier PES fitted to DFT/B3LYP energies and gradients. Ring-polymer instanton calculations of the tunneling splitting of H-atom transfer of tropolone and nine of its isotopomers were performed, including both symmetric and asymmetric tunneling processes. Comparisons with the experiment were made, and (except for one case in which we suspect a misassignment of the experimental data) very good agreement is found for splittings that range from 0.51 to 1.70 cm^–1^. This level of agreement is a significant improvement over results obtained with the DFT-based potential and demonstrates the power of the Δ-machine learned method to extend the process of fitting accurate PESs to molecules with 15 atoms. Mechanistic insights were obtained from the instanton pathways, which indicates the presence of heavy-atom tunneling effects. For the asymmetric isotopomers, the level splitting can be understood in terms of a contribution from the asymmetry of the wells, which changes dramatically with isotopic substitutions, and a contribution from the tunneling, which has only a small dependence on the heavy-atom isotopes. In addition, the instanton path was shown to be “corner cutting”, consistent with the “heavy–light–heavy” kinematics. Thus, this path deviates significantly from the minimum-energy path containing the conventional saddle point and is characteristic of many H-atom transfer kinematics. Future work will be to extend the RPI method to compute the tunneling splittings in vibrationally excited states and compare with experimental measurements.^46^

## Supporting Information Available

Details of the fit to the difference potential, Δ*V*_CC–LL_, a correlation plot of the *V*_LL→CC_ vs direct MTA-CCSD(T) energies, and normal mode analyses at the global minimum and saddle-point of the *V*_LL→CC_ PES; details and results of DF-FNO-CCSD(T) calculations along the instanton pathway (PDF)
