# Supplementary material for: Ring-Polymer Instanton Tunneling Splittings of Tropolone and Isotopomers using a Δ-Machine Learned CCSD(T) Potential: Theory and Experiment Shake Hands
Source: J Am Chem Soc. 2023 Apr 20;145(17):9655–64. doi: 10.1021/jacs.3c00769 (PMC10161208; doi:10.1021/jacs.3c00769)
Supplement: Supplementary file 1 — ja3c00769_si_001.pdf [file ja3c00769_si_001.pdf]

Supporting Information: Ring-Polymer Instanton Tunneling Splittings of Tropolone and Isotopomers using a Δ-Machine Learned CCSD(T) Potential: Theory and Experiment Shake Hands.

Apurba Nandi,[*,†] Gabriel Laude,[‡] Subodh S. Khire,[¶] Nalini D. Gurav,[§] Chen Qu,[∥] Riccardo Conte,[⊥] Qi Yu,[#] Shuhang Li,[†] Paul L. Houston,[*,@] Shridhar R. Gadre,[*,△] Jeremy O. Richardson,[*,‡] Francesco A. Evangelista,[*,†] and Joel M. Bowman[*,†]

†*Department of Chemistry and Cherry L. Emerson Center for Scientific Computation, Emory University, Atlanta, Georgia 30322, U.S.A.*

‡*Laboratory of Physical Chemistry, ETH Zürich, 8093 Zürich, Switzerland.*

¶ *RIKEN Center for Computational Science, Kobe 650-0047,Japan*

§*Organisch-Chemisches Institut, University of Münster, 48149 Münster*

∥*Independent Researcher, Toronto, Canada.*

⊥*Dipartimento di Chimica, Università Degli Studi di Milano, via Golgi 19, 20133 Milano, Italy.*

#*Department of Chemistry Yale University, New Haven, Connecticut 06520, U.S.A.*

@*Department of Chemistry and Chemical Biology, Cornell University, Ithaca, New York 14853, U.S.A. and Department of Chemistry and Biochemistry, Georgia Institute of Technology, Atlanta, Georgia 30332, U.S.A*

△*Department of Scientific Computing, Modelling and Simulation, Savitribai Phule Pune University, Pune 411 007, India.*

E-mail: apurba.nandi@emory.edu; plh2@cornell.edu; gadre@unipune.ac.in; jeremy.richardson@phys.chem.ethz.ch; francesco.evangelista@emory.edu; jmbowma@emory.edu

The Supporting Information contains details of the fit to the difference potential, $\Delta V_{\mathrm{CC-LL}}$, a correlation plot of the $V_{\mathrm{LL}\rightarrow\mathrm{CC}}$ PES vs direct MTA-CCSD(T) energies, and normal mode analyses at the global minimum and saddle-point of the $V_{\mathrm{LL}\rightarrow\mathrm{CC}}$ PES. In addition, we provide details and results of DF-FNO-CCSD(T) calculations along the instanton pathway.

$\Delta V_{\mathrm{CC-LL}}$ and $V_{\mathrm{LL}\rightarrow\mathrm{CC}}$

The difference between MTA-CCSD(T) and DFT energies versus the DFT energies, relative to the DFT minimum are given in Figure S1. (Note although the y-axis is labeled as $\Delta V_{\mathrm{CC-LL}}$ this is not the fit.) The variation in the difference is what we focus on because there is an absolute difference in the electronic energies, say at the global minimum of the DFT-based PES. If there is just a constant energy difference then the DFT and MTA-CCSD(T) PESs

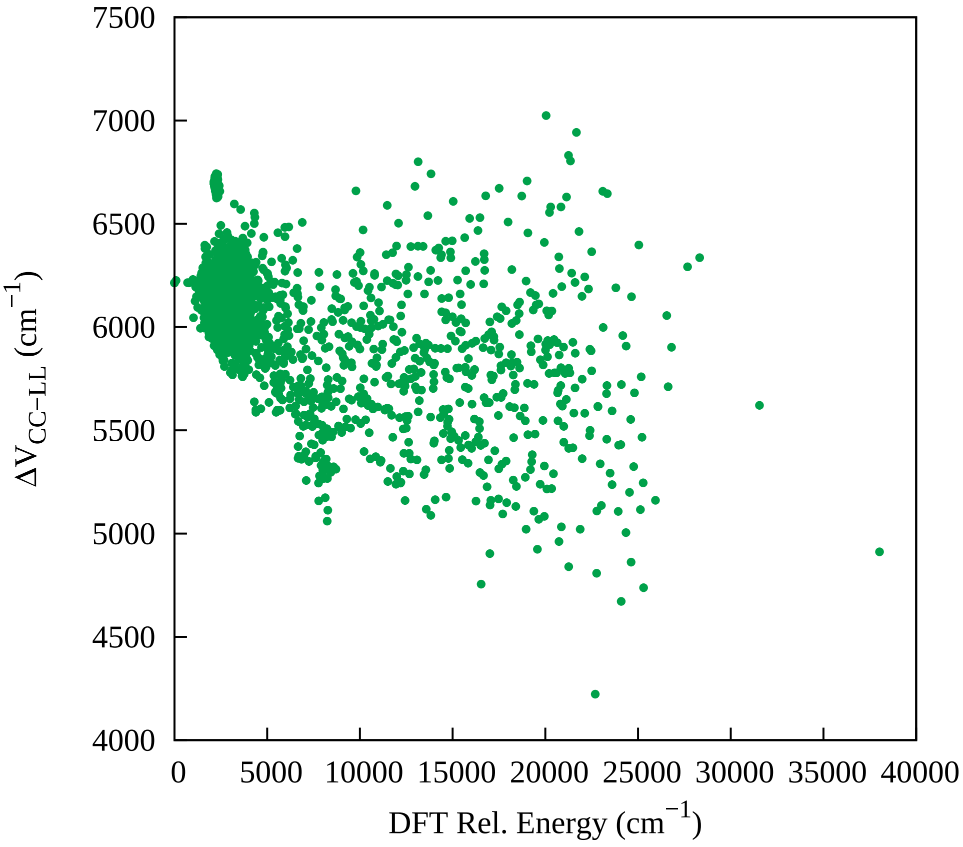

Figure S1: Difference in MTA-CCSD(T) and DFT energies

would be identical. As seen the range of the variation is roughly 2000 cm^{-1}. This is roughly 10 percent of the DFT electronic energies and so fitting this difference can be done with acceptable precision using the low order PIP fitting basis described in the main text.

Ultimately, it is the fidelity of the corrected PES $V_{\text{LL}\to\text{CC}}$ that is of paramount importance. This is examined in Figure S2 where a correlation plot of the $V_{\text{LL}\to\text{CC}}$ PES versus direct MTA-CCSD(T) energies is given. Note the large range in energies and visually good correlation. The RMS error of the PES is roughly 80 cm^{-1} for energies up to 10 000 cm^{-1}, 95 cm^{-1} for energies up to 20 000 and 105 cm^{-1} for all the energies shown in the figure.

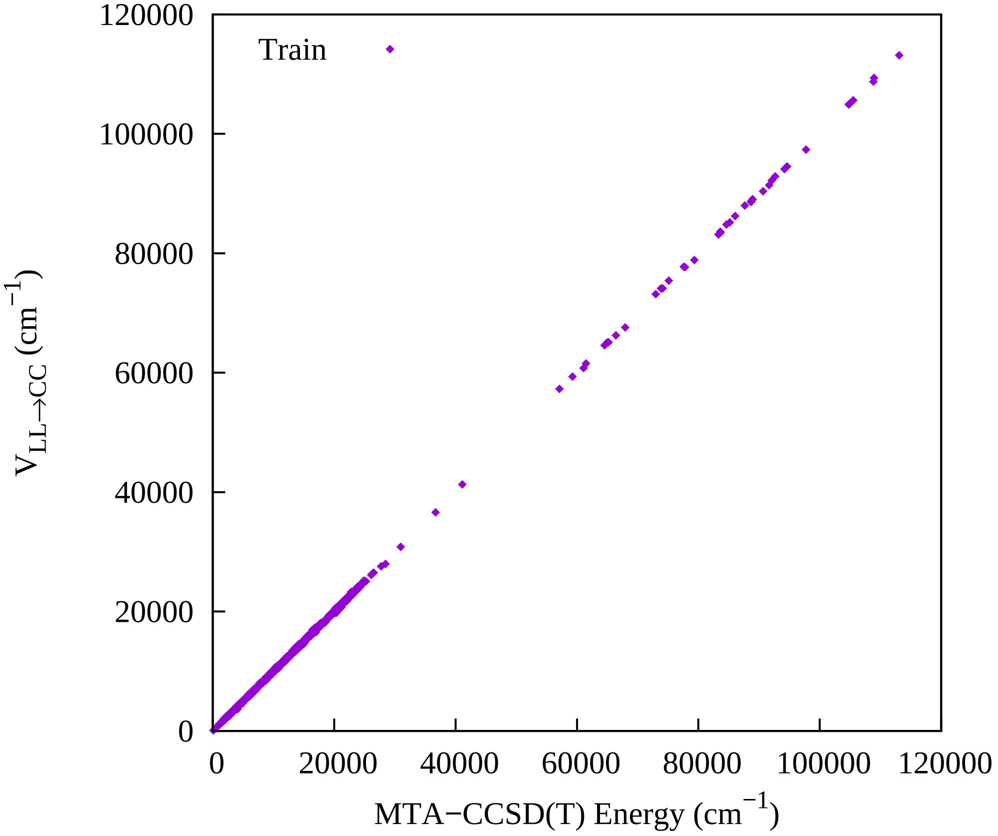

Figure S2: Correlation plot of the corrected PES vs the MTA-CCSD(T) energies

Normal Mode Analyses

Table S1 presents the harmonic frequencies of the corrected PES $V_{\mathrm{LL}\to\mathrm{CC}}$ at the global minimum and the H-atom transfer saddle point.

Table S1: Normal mode frequencies (in cm^{-1}) of the global minimum (GM) and H-transfer saddle point (SP) geometry of Tropolone from the $V_{\mathrm{LL}\to\mathrm{CC}}$ PES. The barrier height of the H-transfer saddle-point is $V^{\ddagger} = 2512\ \mathrm{cm}^{-1}$.

GM				SP			
Mode	Freq.	Mode	Freq.	Mode	Freq.	Mode	Freq.
1	110	21	1039	1	1391*i*	21	1050
2	181	22	1210	2	155	22	1192
3	354	23	1229	3	163	23	1205
4	361	24	1275	4	347	24	1288
5	372	25	1313	5	367	25	1342
6	398	26	1364	6	390	26	1401
7	443	27	1456	7	398	27	1405
8	541	28	1467	8	560	28	1429
9	599	29	1504	9	598	29	1500
10	673	30	1534	10	666	30	1536
11	710	31	1597	11	690	31	1555
12	734	32	1658	12	746	32	1642
13	738	33	1673	13	748	33	1671
14	793	34	3068	14	755	34	2124
15	873	35	3173	15	857	35	3078
16	887	36	3203	16	873	36	3199
17	926	37	3216	17	896	37	3201
18	956	38	3331	18	959	38	3232
19	988	39	3468	19	994	39	3433
20	1016	**	****	20	1005	**	****

DF-FNO-CCSD(T) Calculations

Single-point energies were computed at the coupled cluster with singles, doubles, and perturbative triples [CCSD(T)][1–4] level of theory using the Psi4 *ab initio* quantum chemistry package.[5] These computations employed density fitting (DF)[6,7] to avoid the storage of the four-index electron repulsion integrals and froze core electrons in the evaluation of the post-

Hartree–Fock corrections. We employ the aug-cc-pVTZ-JKFIT[8] auxiliary basis set to generate the Hartree–Fock orbitals and the aug-cc-pVTZ-RI[9,10] auxiliary basis set for the correlation computations. The frozen natural orbital (FNO) technique[11–16] was used to truncate the virtual orbital space and thus reduce the cost of the CCSD(T) computations by a factor of $(V/V_{\mathrm{FNO}})^4$, where V and V_{FNO} represent the number of virtual orbitals before and after FNO truncation, respectively. For the DF-FNO-CCSD(T) procedure, we used a conservative FNO cutoff value of 10^{-6}, which leads to retaining approximately 90% of the virtual orbitals. Benchmark computations show that the impact on the energy barrier of the DF and FNO approximations is negligible (less than 1.5 cm^{-1} and 0.01 cm^{-1}, respectively). With this cutoff value, the DF-FNO-CCSD(T) energy of tropolone may be computed in about 6 hours using 8 threads on a computer node with two Intel Xeon E5-2650 v2 processors and 128 GB of memory. A standard, integral-direct computation of the CCSD(T) energy would require approximately 50 hours on the same computer.

Complete set of instanton results

In Figure S3, we show the potential-energy profile of the instanton paths (according to Δ-ML PES) for H_1 and D_1. In addition, the profile along the minimum-energy pathway (MEP) is shown for the parent isotopomer. The MEP was obtained through the nudged-elastic band approach.[17,18]

In the main text, we employed the 'dual-level method', wherein we re-evaluate the action S at a different level of theory, in order to examine the sensitivity of the Δ-ML PES. We calculated 50 MTA-CCSD(T) and DF-FNO-CCSD(T) points along the Δ-ML instanton path. The potentials of the $N = 1024$ beads along the ring-polymer instanton are linearly interpolated from the 50 calculated points. These rescaled bead potentials are then used to re-evaluate the action with the usual ring-polymer expression.

These points, along with the original Δ-ML instanton path, are shown in Figure S4. Note that no instanton optimization is carried out with the new level of theory. For comparison,

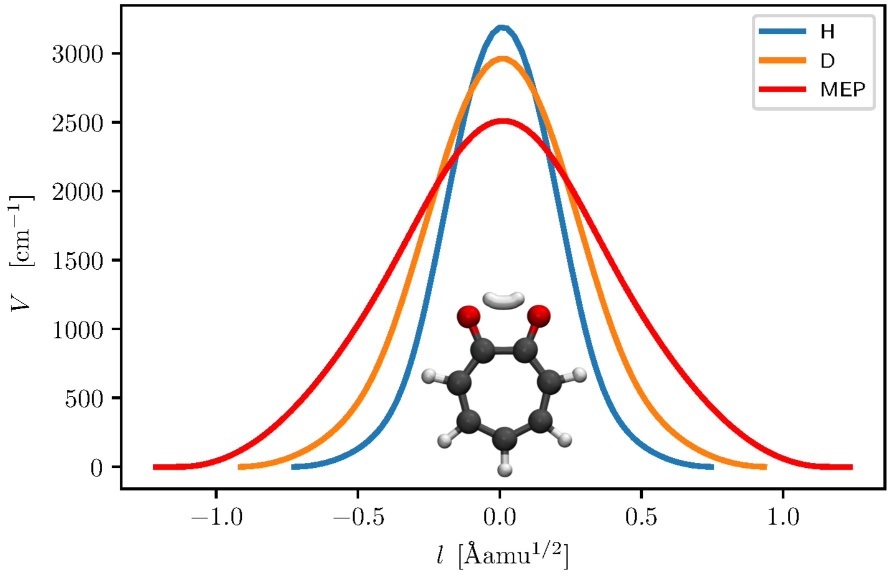

Figure S3: Potential along the instanton as a function of mass-weighted path length l for the parent (i.e. H) and D_1 isotopomer of tropolone. For comparison, the minimum-energy pathway (MEP) for the parent molecule is shown. An atomistic representation of the instanton for the parent molecule is also given.

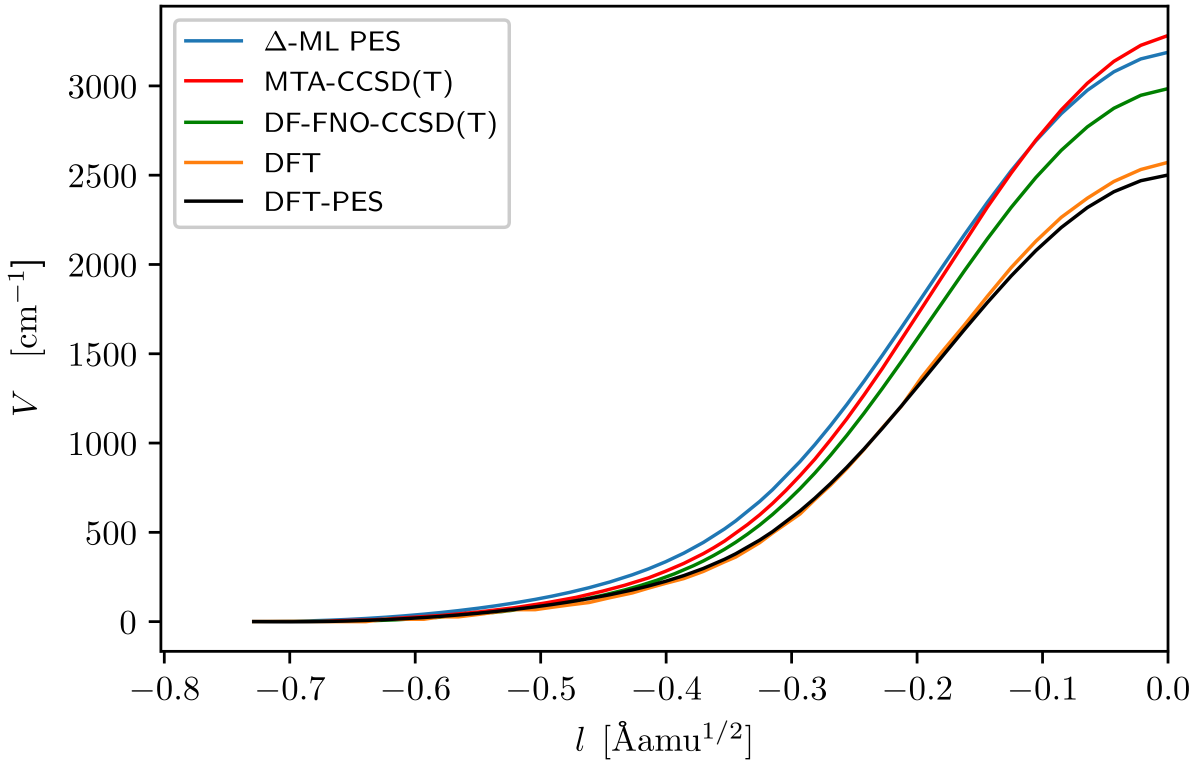

Figure S4: Energies on the Δ-ML instanton path with varying levels of theory, as a function of cumulative mass-weighted path-length l. Here, 'DFT' refers to direct DFT calculations at a B3LYP level of theory, whereas 'DFT-PES' refers to energy calculations done with the DFT-based PES of our previous work presented in Ref. 19. As the barrier is symmetric, only one half is shown.

we additionally show direct DFT calculations at B3LYP level of theory as well as calculations on the PES developed in our previous work.[19] In Table S2 we present the energy difference between the instanton barrier top and bottom, evaluated through various levels of theory including DFT from the DFT PES instanton path and direct DFT along the Δ-ML instanton path. In the same table, we also present the recalculated action S for different levels of theory.

We also evaluated the action $S = \int \sqrt{2V(l)}\, \mathrm{d}l$ along the minimum-energy path shown in Figure S3. This gives $S/\hbar = 15.67$, which implies that neglecting corner cutting will result in a significant underestimation of the true splitting.

Combining the recalculated action S along with an unmodified pre-exponential factor

Table S2: Difference in energy between the instanton barrier top and bottom, $V^{\ddagger}_{\text{inst}}$, calculated with different levels of theory for the parent isotopomer. In addition, the recalculated action, S, for different levels of theory is given where available. 'DFT' here refers to direct DFT calculations at a B3LYP level of theory. 'DFT-PES' refers to the PES presented in Ref. 19. Unless otherwise noted, all values were evaluated using an instanton path optimized on the Δ-ML PES.

Level of theory	$V^{\ddagger}_{\text{inst}}$ [cm^{-1}]	$S/\hbar$
Δ-ML PES	3188	9.49
MTA-CCSD(T)	3281	9.19
DF-FNO-CCSD(T)	2985	8.76
CCSD(T)/aVTZ	2987	-
CCSD(T)-F12/aVTZ	3079	-
DFT	2572	8.17
DFT-PES	2501	8.19
DFT-PES[a]	2561	8.24

[a]Instanton path optimized on the DFT PES of Ref. 19.

A, we evaluated $\Omega = A \exp(-S/\hbar)$ and therefore the level splitting Δ corresponding to the desired level of theory. This data is presented in Table S3, as well as in a pictorial manner in Figure S5.

It can be seen that the instanton results corrected by DF-FNO-CCSD(T) overpredict the experimental results by about 40%. If we were to fully optimize the instanton on the DF-FNO-CCSD(T) surface, the action would decrease, thus causing the splittings to increase further. As discussed in the main text, we assume that this discrepancy is the result of an incomplete basis set. The fact that the explicitly-correlated CCSD(T)-F12/aVTZ barrier height, $V^{\ddagger}_{\text{inst}}$, is higher than for CCSD(T)/aVTZ lends weight to this assumption.

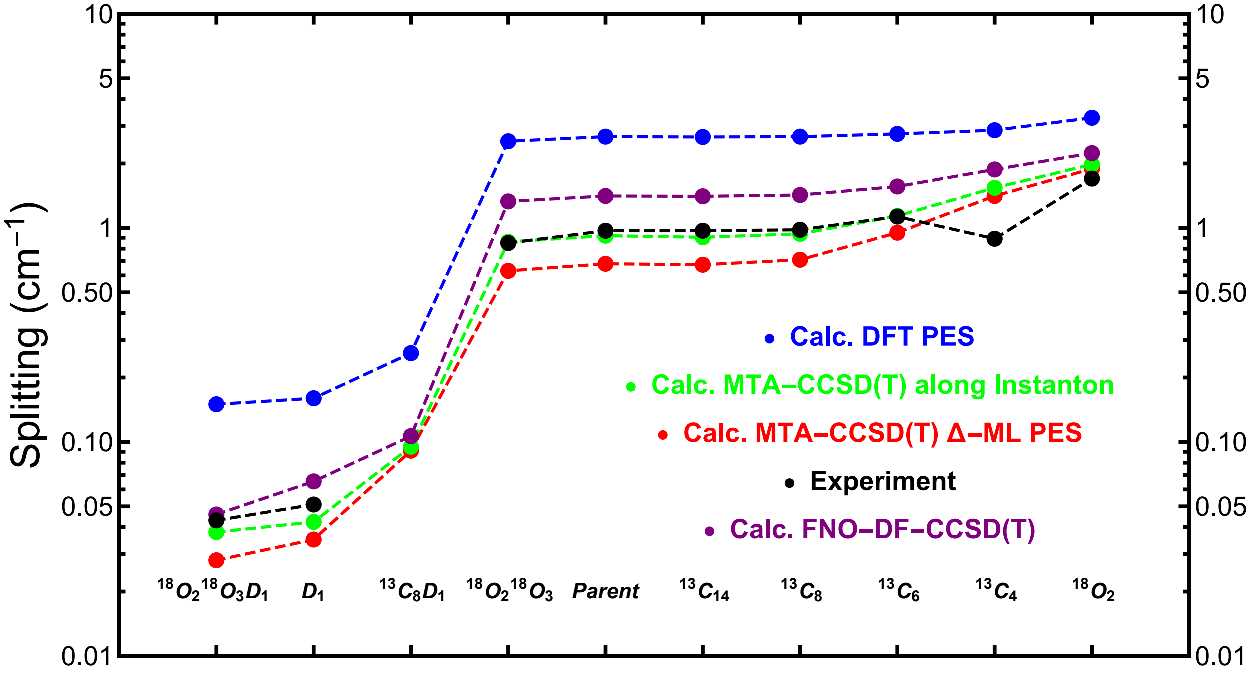

Figure S5: Comparison of Experimental and Calculated Tunneling Splittings. Blue, red, green and purple data indicate the Instanton results using the DFT PES, the Δ-ML MTA-CCSD(T) PES, MTA-CCSD(T), and DF-FNO-CCSD(T) calculations along the instanton path, respectively. The black data indicate the experimental results. Isotopomers are listed at the bottom, where the subscript numbers indicate the atom(s) that were isotopically substituted, using the numbering from Figure 1 of the main text.

Table S3: A collection of all splittings Δ for all isotopomers, including all correction schemes. Here, the level of theory of the correction is marked by the superscript, e.g. $\Delta_{\Delta\text{-ML}}^{\text{DF-FNO-CCSD(T)}}$ refers to the splitting calculated wherein a few points along the Δ-ML instanton were recalculated at a DF-FNO-CCSD(T) level of theory.

Isotopomer	Δ_{DFT}	$\Delta_{\Delta\text{-ML}}$	$\Delta_{\Delta\text{-ML}}^{\text{MTA-CCSD(T)}}$	$\Delta_{\Delta\text{-ML}}^{\text{DF-FNO-CCSD(T)}}$	$\Delta_{\text{expt.}}$
Parent	2.67	0.68	0.92	1.42	0.97 [20,21]
$^{13}C_{14}$	2.66	0.67	0.91	1.41	0.97 [21]
$^{18}O_2{}^{18}O_3$	2.54	0.63	0.86	1.33	0.83, [22] 0.83 [23]
D_1	0.16	0.031	0.042	0.065	0.051 [21,23]
$^{18}O_2{}^{18}O_3D_1$	0.15	0.028	0.038	0.059	0.043 (estimated) [23]
$^{18}O_2$	3.27	1.89	1.98	2.24	1.70 (estimated) [22,24]
$^{13}C_4$	2.86	1.41	1.54	1.88	0.89 (outlier) [21]
$^{13}C_6$	2.75	0.95	1.14	1.56	1.13 [21]
$^{13}C_8$	2.67	0.71	0.94	1.42	0.98 [21]
$^{13}C_8D_1$	0.26	0.091	0.095	0.11	–

References

(1) Raghavachari, K.; Trucks, G. W.; Pople, J. A.; Head-Gordon, M. A Fifth-Order Perturbation Comparison of Electron Correlation Theories. *Chem. Phys. Lett.* **1989**, *157*, 479–483.

(2) Bartlett, R. J.; Watts, J.; Kucharski, S.; Noga, J. Non-Iterative Fifth-Order Triple and Quadruple Excitation Energy Corrections in Correlated Methods. *Chem. Phys. Lett.* **1990**, *165*, 513–522.

(3) Deegan, M. J.; Knowles, P. J. Perturbative Corrections to Account for Triple Excitations in Closed and Open Shell Coupled Cluster Theories. *Chem. Phys. Lett.* **1994**, *227*, 321–326.

(4) Stanton, J. F. Why CCSD(T) Works: A Different Perspective. *Chem. Phys. Lett.* **1997**, *281*, 130–134.

(5) Smith, D. G.; Burns, L. A.; Simmonett, A. C.; Parrish, R. M.; Schieber, M. C.; Galvelis, R.; Kraus, P.; Kruse, H.; Di Remigio, R.; Alenaizan, A., et al. PSI4 1.4: Open-source software for high-throughput quantum chemistry. *J. Chem. Phys.* **2020**, *152*, 184108.

(6) Whitten, J. L. Coulombic potential energy integrals and approximations. *J. Chem. Phys.* **1973**, *58*, 4496–4501.

(7) Dunlap, B. I.; Connolly, J.; Sabin, J. On some approximations in applications of Xα theory. *J. Chem. Phys.* **1979**, *71*, 3396–3402.

(8) Weigend, F. A fully direct RI-HF algorithm: Implementation, optimised auxiliary basis sets, demonstration of accuracy and efficiency. *Phys. Chem. Chem. Phys.* **2002**, *4*, 4285–4291.

(9) Weigend, F.; Köhn, A.; Hättig, C. Efficient use of the correlation consistent basis sets in resolution of the identity MP2 calculations. *J. Chem. Phys.* **2002**, *116*, 3175–3183.

(10) Hättig, C. Optimization of auxiliary basis sets for RI-MP2 and RI-CC2 calculations: Core–valence and quintuple-ζ basis sets for H to Ar and QZVPP basis sets for Li to Kr. *Phys. Chem. Chem. Phys.* **2005**, *7*, 59–66.

(11) Sosa, C.; Geertsen, J.; Trucks, G. W.; Bartlett, R. J.; Franz, J. A. Selection of the reduced virtual space for correlated calculations. An application to the energy and dipole moment of H_2O. *Chem. Phys. Lett.* **1989**, *159*, 148–154.

(12) Klopper, W.; Noga, J.; Koch, H.; Helgaker, T. Multiple basis sets in calculations of triples corrections in coupled-cluster theory. *Theor. Chem. Acc.* **1997**, *97*, 164–176.

(13) Taube, A. G.; Bartlett, R. J. Frozen natural orbitals: Systematic basis set truncation for coupled-cluster theory. *Collect. Czech. Chem. Commun* **2005**, *70*, 837–850.

(14) Landau, A.; Khistyaev, K.; Dolgikh, S.; Krylov, A. I. Frozen natural orbitals for ionized states within equation-of-motion coupled-cluster formalism. *J. Chem. Phys.* **2010**, *132*, 014109.

(15) DePrince III, A. E.; Sherrill, C. D. Accurate noncovalent interaction energies using truncated basis sets based on frozen natural orbitals. *J. Chem. Theory Comput.* **2013**, *9*, 293–299.

(16) DePrince III, A. E.; Sherrill, C. D. Accuracy and efficiency of coupled-cluster theory using density fitting/Cholesky decomposition, frozen natural orbitals, and at 1-transformed Hamiltonian. *J. Chem. Theory Comput.* **2013**, *9*, 2687–2696.

(17) Henkelman, G.; Uberuaga, B. P.; Jónsson, H. A climbing image nudged elastic band method for finding saddle points and minimum energy paths. *J. Chem. Phys.* **2000**, *113*, 9901–9904.

(18) Sheppard, D.; Terrell, R.; Henkelman, G. Optimization methods for finding minimum energy paths. *J. Chem. Phys.* **2008**, *128*, 134106.

(19) Houston, P.; Conte, R.; Qu, C.; Bowman, J. M. Permutationally invariant polynomial potential energy surfaces for tropolone and H and D atom tunneling dynamics. *J. Chem. Phys.* **2020**, *153*, 024107.

(20) Tanaka, K.; Honjo, H.; Tanaka, T.; Kohguchi, H.; Ohshima, Y.; Endo, Y. Determination of the proton tunneling splitting of tropolone in the ground state by microwave spectroscopy. *J. Chem. Phys.* **1999**, *110*, 1969–1978.

(21) Keske, J. C.; Lin, W.; Pringle, W. C.; Novick, S. E.; Blake, T. A.; Plusquellic, D. F. High-resolution studies of tropolone in the S_0 and S_1 electronic states: Isotope driven dynamics in the zero-point energy levels. *J. Chem. Phys.* **2006**, *124*, 074309.

(22) Redington, R. L.; Redington, T. E.; Blake, T. A.; Sams, R. L.; Johnson, T. J. ^{18}O Effects on the Infrared Spectrum and Skeletal Tunneling of Tropolone. *J. Chem. Phys.* **2005**, *122*, 224311.

(23) Redington, R. L.; Redington, T. E.; Sams, R. L. Infrared Absorption Spectra in the Hydroxyl Stretching Regions of Gaseous Tropolone OHO Isotopomers. *Z. Phys. Chem.* **2008**, *222*, 1197–1211.

(24) Redington, R. L.; Redington, T. E.; Sams, R. L. Tunneling Splittings for "O$\cdots$O Stretching" and Other Vibrations of Tropolone Isotopomers Observed in the Infrared Spectrum Below 800 cm^{-1}. *J. Phys. Chem. A* **2008**, *112*, 1480–1492.
